# Lung recruitment induced by Sigh in hypoxemic intubated critically ill patients

**DOI:** 10.1186/cc12042

**Published:** 2013-03-19

**Authors:** TM Mauri, G Bellani, A Coppadoro, P Tagliabue, A Barletta, V Meroni, M Teggia Droghi, N Patroniti, A Pesenti

**Affiliations:** 1 University of Milan-Bicocca, Monza, Italy; 2 San Gerardo Hospital, Monza, Italy

## Introduction

In intubated critically ill patients, cyclic short recruitment manoeuvres (Sigh) introduced during pressure support ventilation (PSV) improve oxygenation, probably by increasing end-expiratory lung volume (EELV). We assessed Sigh effects on regional distribution of EELV by electrical impedance tomography (EIT): a bedside, radiation-free lung imaging technique. Moreover, we investigated baseline characteristics correlated with response to Sigh.

## Methods

We enrolled 20 intubated critically ill patients undergoing PSV with PaO_2_/FiO_2 _≤300 mmHg and PEEP ≥5 cmH_2_O. We applied on each patient's thorax a 16-electrode belt connected to an EIT monitor (PulmoVista 500^®^; Dräger Medical GmbH, Lübeck, Germany). Sigh was introduced as 35 cmH_2_O continuous positive airway pressure phase lasting 3 to 4 seconds at different rates (0, 0.5, 1, 2 per minute, random order) for 20 minutes. From raw EIT data, we calculated global changes in EELV (ΔEELVgl), calculated as changes in end-expiratory lung impedance calibrated versus tidal volume to estimate changes in ΔEELV and considering 0 Sigh/minute as baseline; and ΔEELV of nondependent and dependent lung regions (ΔEELVnondep and ΔEELVdep). Together, we collected ventilation parameters, hemodynamics and arterial blood gases.

## Results

Patients were 65 ± 13 years old, ventilation days were 9 ± 10 and PaO_2_/FiO_2 _was 194 ± 49 mmHg at PEEP 8 ± 2 cmH_2_O. Introduction of Sigh improved PaO_2_/FiO_2 _(*P <*0.001) and ΔEELVgl (*P <*0.001). The increase in EELV was diffuse across all lung regions as both ΔEELVnondep and ΔEELVdep changed (*P <*0.01 and *P *= 0.06). Minute ventilation, arterial blood pH and hemodynamics were not significantly affected by introduction of Sigh, while mean airway pressure increased (*P <*0.001), albeit only at the highest Sigh rate and by a limited extent (~2 cmH_2_O). Patients with mean ΔEELVgl over the three Sigh phases >200 ml (that is, Sigh-responders) had significantly lower PaO_2_/FiO_2 _values at baseline (that is, at Sigh rate = 0/minute) than Sighnonresponders (163 ± 25 mmHg vs. 222 ± 39 mmHg, *P <*0.01).

## Conclusion

Introduction of Sigh in intubated critically ill patients undergoing PSV improves gas exchange by inducing alveolar recruitment across all lung regions with minimal increase of airway pressure. Patients with more severe gas exchange impairment present the highest degree of potentially recruitable lung.

